# Coordinate regulation of *ELF5* and *EHF* at the chr11p13 CF modifier region

**DOI:** 10.1111/jcmm.14646

**Published:** 2019-09-26

**Authors:** Hannah Swahn, Jey Sabith Ebron, Kay‐Marie Lamar, Shiyi Yin, Jenny L. Kerschner, Monali NandyMazumdar, Candice Coppola, Eric M. Mendenhall, Shih‐Hsing Leir, Ann Harris

**Affiliations:** ^1^ Department of Genetics and Genome Sciences Case Western Reserve University Cleveland OH USA; ^2^ Department of Biological Sciences University of Alabama in Huntsville Huntsville AL USA

**Keywords:** airway epithelial biology, chromatin architecture, *cis‐*regulatory elements, E74‐like factor 5, enhancers, ETS family transcription factors, ETS‐homologous factor, lung diseases, lung epithelium, regulation of gene expression

## Abstract

E74‐like factor 5 (*ELF5*) and ETS‐homologous factor (*EHF*) are epithelial selective ETS family transcription factors (TFs) encoded by genes at chr11p13, a region associated with cystic fibrosis (CF) lung disease severity. EHF controls many key processes in lung epithelial function so its regulatory mechanisms are important. Using CRISPR/Cas9 technology, we removed three key *cis*‐regulatory elements (CREs) from the chr11p13 region and also activated multiple open chromatin sites with CRISPRa in airway epithelial cells. Deletion of the CREs caused subtle changes in chromatin architecture and site‐specific increases in *EHF* and *ELF5*. CRISPRa had most effect on *ELF5* transcription. ELF5 levels are low in airway cells but higher in LNCaP (prostate) and T47D (breast) cancer cells. ATAC‐seq in these lines revealed novel peaks of open chromatin at the 5’ end of chr11p13 associated with an expressed *ELF5* gene. Furthermore, 4C‐seq assays identified direct interactions between the active *ELF5* promoter and sites within the *EHF* locus, suggesting coordinate regulation between these TFs. ChIP‐seq for ELF5 in T47D cells revealed ELF5 occupancy within *EHF* introns 1 and 6, and siRNA‐mediated depletion of ELF5 enhanced *EHF* expression. These results define a new role for ELF5 in lung epithelial biology.

## 1 INTRODUCTION

Many monogenic disorders have significant phenotypic variability that may be attributed to genetic elements outside the causative locus. These sites (modifier loci) are often identified by genome‐wide‐association studies (GWAS).^1^ However, defining the mechanisms whereby modifier loci contribute to phenotype is often challenging.^2^ One example is cystic fibrosis (CF) a life‐shortening, recessive genetic disease caused by mutations in the cystic fibrosis transmembrane conductance regulator (*CFTR)* gene. Of note, mutations in *CFTR* do not correlate with lung disease severity, the major cause of morbidity in CF.^3^, ^4^, ^5^

CF impacts about 70,000 people worldwide 6 though phenotype does not correlate well with mutations in *CFTR*. The high frequency of the disorder facilitated a replicated GWAS, which identified single nucleotide polymorphisms (SNPs) in an intergenic region of chromosome 11p13 that significantly associated with CF lung disease severity.^3^, ^4^ The genes mapping closest to these SNPs are two epithelial–selective E twenty‐six (ETS) transcription factors (TFs), ETS homologous factor (*EHF*) and E74‐like factor 5 (*ELF5*) on the 5’ side and Apaf‐1‐interacting protein (*APIP*) on the 3’ side.

*EHF* is abundantly expressed in differentiated epithelial tissues, particularly in the prostate, pancreas, salivary gland and airway.^7^, ^8^ EHF can be a transcriptional activator or repressor, depending on cellular context, and its expression is often reduced or lost in some breast, prostate and lung carcinomas.^7^, ^8^, ^9^, ^10^ In the airway, EHF is intimately involved in many cellular processes integral to CF lung pathology such as epithelial cell differentiation and development, cell locomotion, response to wounding, barrier function, goblet cell differentiation, tissue remodelling and inflammation.^11^, ^12^ Also, EHF has a direct role in *CFTR* regulation, as it binds to an airway selective enhancer element of *CFTR* and represses its expression.^13^ For these reasons, *EHF* is a strong candidate CF modifier gene and also a potential CF therapeutic target since elevated CFTR substrate may enhance the efficacy of correctors and potentiators.^14^

In contrast, the role of ELF5 has been studied in breast cancer, where it drives lung metastasis by recruiting myeloid‐derived suppressor cells.^15^ ELF5 is also necessary for proper lung branching and epithelial differentiation in the mouse.^16^ It has not been well studied in the human lung, although it is a candidate gene for conferring susceptibility to asthma.^17^ Furthermore, ELF5 was predicted to regulate *EHF* expression, since ChIP‐seq with an antibody specific for ELF5 showed its occupancy at the *EHF* promoter and first intron in the breast cancer cell line T47D.^18^

Although to date, the highest p‐value SNPs identified in the replicated GWAS do not correlate with the transcription of genes at 11p13, this may reflect a paucity of data on gene expression at the single‐cell level. However, revealing the regulatory interactions of the EHF and ELF5 TFs is critical to understanding their role in lung epithelial biology. Here we investigate the *cis*‐regulatory elements (CREs) at chr11p13 that may control the expression of these two epithelial selective ETS transcription factors. Using CRISPR/Cas9 technology, three key CREs were deleted from the region in airway epithelial cells, and subsequent changes in gene expression and chromatin architecture were examined. We also took an unbiased screening approach to identify additional CREs at chr11p13 by activating multiple DNase I hypersensitive sites (DHS) across the region using CRISPRa,^19^ followed by measuring nearby gene expression. In parallel, we pursued the predicted role of ELF5 in regulating *EHF* by investigating open chromatin and 3D architecture in cell types with different abundance of *ELF5*. These genomic context studies revealed a critical role for ELF5 in coordinating the complex regulatory environment at chr11p13. Our results are relevant to the involvement of both ELF5 and EHF in lung disease, since both genes are expressed in human bronchial epithelium.

## 2 MATERIALS AND METHODS

### 2.1 Cell culture

A549,^20^ A549 c9 (subclone of A549 that endogenously expresses EHF protein at levels higher than WT),^11^ Calu3 21 and 16HBE14o‐ 22 cells were grown in Dulbecco's modified Eagle's medium (low glucose) with 10% foetal bovine serum (FBS). LNCaP and T47D cells were grown in Roswell Park Memorial Institute (RPMI) 1640 medium supplemented with 10% FBS 23 and for T47D only, 0.2 Units/mL insulin.^24^

### 2.2 Luciferase‐based reporter assays

Luciferase promoter:enhancer constructs were generated as described previously.^25^ Constructs were transfected with Lipofectamine 2000 (Life Technologies) into A549 c9 and T47D cells. A modified pRL Renilla luciferase vector (Promega) was used as a transfection control. Cells were assayed for Renilla and firefly luciferase activity after 48 hours using the Dual‐Luciferase Reporter Assay System (Promega).^26^

### 2.3 CRISPR guide design, CRISPR/Cas9 transfection and screening

Two pairs of gRNAs flanking *EHF* intron 6, 11.2521 and 11.2516 were identified using Benchling 27 (Table S1). Both gRNAs flanking each region were sequentially cloned into a single pBlueScript (pBS) with a modified multiple cloning site. A549 c9 cells were seeded onto 6‐well plates and transfected after 24 hours with 0.1 pmol of pMJ920 (wild‐type Cas9 plasmid tagged with GFP) (Addgene, plasmid #42234) and 0.2 pmol of pBS containing the 5’ and 3’ gRNAs using Lipofectamine 2000 (Life Technologies); 48 hours after transfection, cells were prepared for fluorescence‐activated cell sorting of GFP‐positive cells and single cells were plated onto 96‐well plate. Clones were expanded and screened for homozygous deletion of each site as described previously using primers shown in Table S2.^28^

### 2.4 Reverse transcription quantitative polymerase chain reaction (RT‐qPCR)

RNA was extracted from cells using TRIzol (In Vitrogen) according to manufacturer's instructions. Reverse transcription used TaqMan Reverse Transcription Reagents (Life Technologies) by the standard protocol. qPCR assays were performed using SYBR Green reagents (Life Technologies) and primers listed in Table S3.

### 2.5 Chromatin immunoprecipitation (ChIP)

Chromatin immunoprecipitation (ChIP)‐seq was performed as previously described 11 with an antibody specific for ELF5 (Santa Cruz, sc‐376737). ChIP‐qPCR was performed as described earlier.^29^, ^30^ Antibodies were specific for CTCF (Millipore 07‐729) or normal rabbit IgG (Millipore 12‐370). qPCR was performed using SYBR Green reagents with primers listed in Table S4.

### 2.6 Circular chromosome conformation capture and deep sequencing (4C‐seq)

4C‐seq was performed as previously described 28 in A549 c9 and its derivative CRE deletion clones, Calu3, LNCaP and T47D cells. The primers used to generate libraries for each viewpoint are listed in Table S5. Two independent 4C libraries were generated for each viewpoint for each cell type. The sequencing data were processed using the 4Cseqpipe pipeline.^31^ All 4C‐seq domainograms were generated using the default parameters of the pipeline.

### 2.7 CRISPRa/VPR‐mediated activation experiments

Single guide RNAs (sgRNAs) targeting *ELF5* and *EHF* promoters were designed within 1 kb 5’ to the transcriptional start sites (TSS). Two to four sgRNAs were designed to target DHS cores at chr11p13 using Benchling 27 (Table S6) and were chosen based on the highest on‐target and lowest off‐target scores. sgRNAs were cloned into the pSPgRNA plasmid (Addgene #47108) and nucleofected with the SP‐dCas9‐VPR plasmid (Addgene #63798) into either 16HBE14o‐ or A549 cells with Lonza 4D‐Nucleofector kits (P3 Primary Cell V4XP‐3032 for 16HBE14o‐ and SE Cell Line V4XC‐1032 for A549). For each experiment, pSPgRNA alone was nucleofected with SP‐dCas9‐VPR and used as a negative control. Using protocols described above, RNA was extracted 48 hours post‐nucleofection and reverse transcription quantitative polymerase chain reaction (RT‐qPCR) assays were performed. Data were normalized individually to the empty vector control.

### 2.8 Omni Assay for transposase accessible chromatin and deep sequencing

Omni‐ATAC‐seq was performed on 50,000 Calu3, LNCaP and T47D cells as described previously^32^ with minor modifications. ATAC‐seq libraries were purified with Agencourt AMPure XP magnetic beads (Beckman Coulter) with a sample to bead ratio of 1:1.2 and eluted in Buffer EB (Qiagen). Data were processed by the ENCODE‐DCC/atac‐seq‐pipeline.

### 2.9 Transient ELF5 siRNA depletion experiments

ELF5 depletion in T47D cells was performed using Silencer select siRNA (Ambion, AM16708) as previously described.^11^ Using protocols described above, RNA was extracted 48 hours post‐transfection and RT‐qPCR assays were performed. For protein assays, lysates were analysed by standard western blot methods.^11^, ^12^ Antibodies were specific for EHF (Clone 5A.5, kindly donated by Dr A Tugores) and β‐tubulin (T4026, Sigma‐Aldrich).

### 2.10 Statistical analysis

Error bars denote standard deviation (SD) or standard error of the mean (SEM) as noted in figure legends, which also define the statistical analysis used. This was either two‐way ANOVA plus multiple comparisons tests or Student's unpaired *t* tests. All statistical analysis was performed in Prism software (GraphPad).

## 3 RESULTS

### 3.1 Deletion of a CRE in *EHF* intron 6 enhances *EHF* expression while impairing recruitment of a strong enhancer to the gene promoter

**Figure 1:**
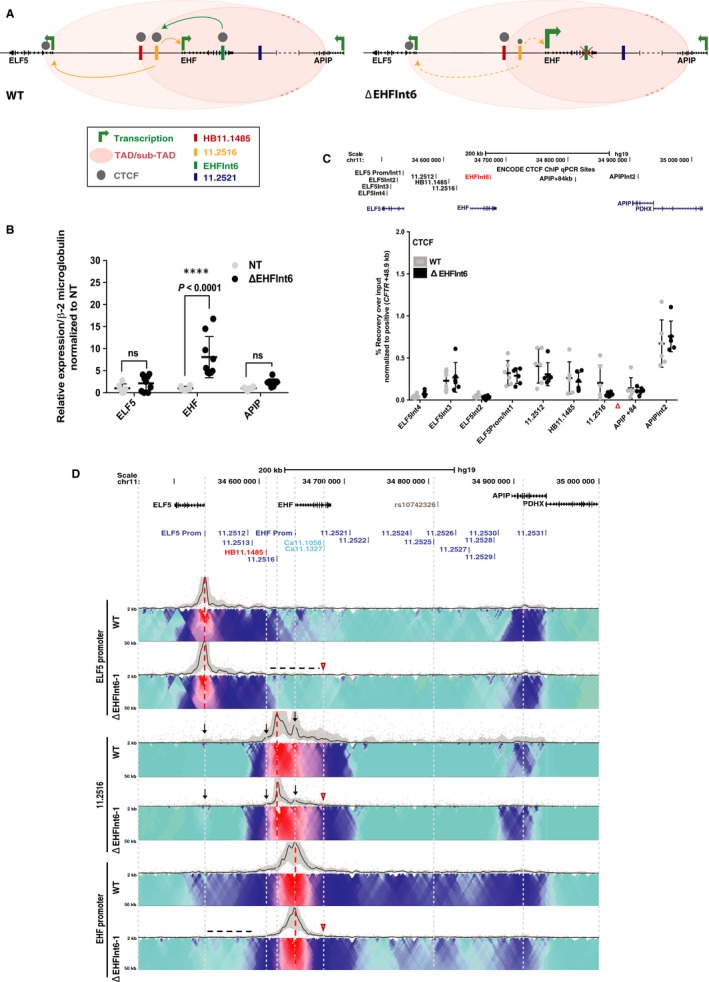
Impact of deletion of *cis*‐element *EHF* intron 6 in A549 c9 cells. (A) *EHF* intron 6 maintains a structural role at 11p13 and helps to facilitate looping of the 11.2516 enhancer (green arrow) to the *ELF5* and *EHF* promoters (yellow arrows). Upon deletion of *EHF* intron 6 (red X), *EHF* expression is enhanced. Additionally, CTCF occupancy at 11.2516 is reduced (smaller grey circle) and its interactions with the *ELF5* and *EHF* promoters are weakened (yellow dashed arrows). (B) RT‐qPCR for *ELF5*, *EHF* and *APIP* expression in non‐targeted WT (NT) and deletion clones. Data are normalized to β2M and relative to clonal WT. Error bars are standard deviation (SD), n ≥ 3. ****P < .0001, ***P < .001, **P < .01, *P < .05, ns = not significant by a two‐way ANOVA test with multiple comparisons. (C) ChIP‐qPCR for CTCF enrichment at interacting sites across the 11p13 region in WT and deletion clones. ChIP results are shown as percent recovery over input and are normalized to a positive control (*CFTR* + 48.9 kb (28). Error bars are SD, n ≥ 2. (D) Circular chromosome conformation capture (4C‐seq) data in WT and one *EHF* intron 6 deletion clone. This deletion removes the Ca11.1058 and Ca11.1327 DHS. Viewpoints at the *ELF5* promoter, 11.2516 and *EHF* promoters are shown (red dotted lines). Open red arrowheads indicate the deletion site. The upper panel (black line) shows the main trend of interaction, and the colour‐coded domainogram indicates relative interactions with a window size ranging from 2‐50kb. Red denotes the strongest interactions; dark blue, to turquoise, to grey represent decreasing interaction frequencies. Informative interactions or loss thereof are shown as black dotted lines or black arrows

The *EHF* locus coincides with an extended track of the H3K27ac active histone mark, indicative of a stretch enhancer in airway epithelial cells.^25^, ^33^ *EHF* intron 6 lies at the 3’ end of this feature and coincides with a region of high occupancy by multiple transcription factors (ENCODE data 34, 35, 36). To determine the role of this element in the genomic context within airway epithelial cells, CRISPR/Cas9 was used to generate A549 c9 clones lacking an ~3.1 kb fragment coinciding with the extent of TF occupancy. Three homozygous deletion clones were evaluated and compared both to non‐targeted clones from the same experiment and wild‐type (WT) A549 c9 cells. Models of the 11p13 region (left) and the impact of removal of *EHF* intron 6 (right) are shown in Figure 1A. First, expression of the *ELF5*, *EHF* and *APIP* genes was assayed by RT‐qPCR (Figure 1B). Removal of the *EHF* intron 6 element did not change *ELF5* or *APIP* transcript levels; however, it was associated with an increase in *EHF* (~8‐fold, *P* < .0001) expression.

These results suggest that either transcriptional repressors of *EHF* are recruited to the intron 6 element or that loss of this site alters chromatin architecture at the locus, thus facilitating occupancy of activating factors at the *EHF* promoter or its enhancers. To investigate the latter hypothesis, we first assayed CTCF occupancy at specific sites across the chr11p13 region by ChIP‐qPCR. CCCTC‐binding factor (CTCF) is an architectural protein involved in higher order chromatin organization. It is recruited to invariant sites in the genome (topologically associated domain (TAD) boundaries) in addition to variant structural elements that may be cell‐type specific.^37^, ^38^, ^39^ The *EHF* intron 6 element binds CTCF with high affinity in several airway cell types,^25^ so its removal was predicted to impact CTCF recruitment at adjacent sites. Consistent with this suggestion, deletion of the *EHF* intron 6 element was associated with a substantial (~3‐fold) decrease in CTCF occupancy at the adjacent site DHS11.2516 (Figure 1C), though this did not reach statistical significance.

To examine the impact of loss of the *EHF* intron 6 element on 3D chromatin structure more broadly, we performed 4C‐seq on the deletion clones and compared them to WT A549 c9 cells (Figure 1D). A minimum of 2 deletion clones were analysed for each viewpoint, and each clone was evaluated in replica experiments. One experiment is shown in each 4C‐seq panel and is consistent with its replicates. For each 4C‐seq panel, the black line shows the main trend of interactions across the locus while below it is the domainogram,^40^ which uses colour‐coded intensity values to show relative interactions with window sizes varying from 2 to 50 kb.^31^ Red denotes the strongest interactions and dark blue, through turquoise, to grey represent gradually decreasing frequencies. Black arrows or dotted lines denote important data features, and the location of the *EHF* intron 6 element deletion is shown by an open red arrowhead. Here, we focus on the gain or loss of interactions at sites we showed previously to have functional importance,^25^ specifically those at the 5’ end of the 11p13 modifier region. Using a viewpoint at the *ELF5* promoter, the deletion is seen to cause a general loss of interactions 3’ to the HB11.1485 CTCF site (black dotted line), most notably close to 11.2516 and within the first intron of *EHF*. With a viewpoint located 20kb 5’ to the *EHF* promoter (11.2516), interactions with the *ELF5* and *EHF* promoters were notably reduced and those with HB11.1485 element were slightly diminished (black arrows). These results are consistent with our earlier data from WT cells showing that DHS11.2516 and the *EHF* intron 6 element interact directly.^25^ The *EHF* promoter viewpoint shows a modest loss of interactions across the whole region, most prominently between HB11.1485 and the *ELF5* promoter (black dotted line). In summary, these data suggest that looping of the CRE at DHS11.2516 to the *EHF* and *ELF5* promoters is somewhat dependent on the *EHF* intron 6 element and moreover that this element contributes to the maintenance of locus architecture.

### 3.2 Deletion of the 11.2521 CRE enhances *ELF5* expression and increases interactions of the *ELF5* promoter with novel sites

**Figure 2:**
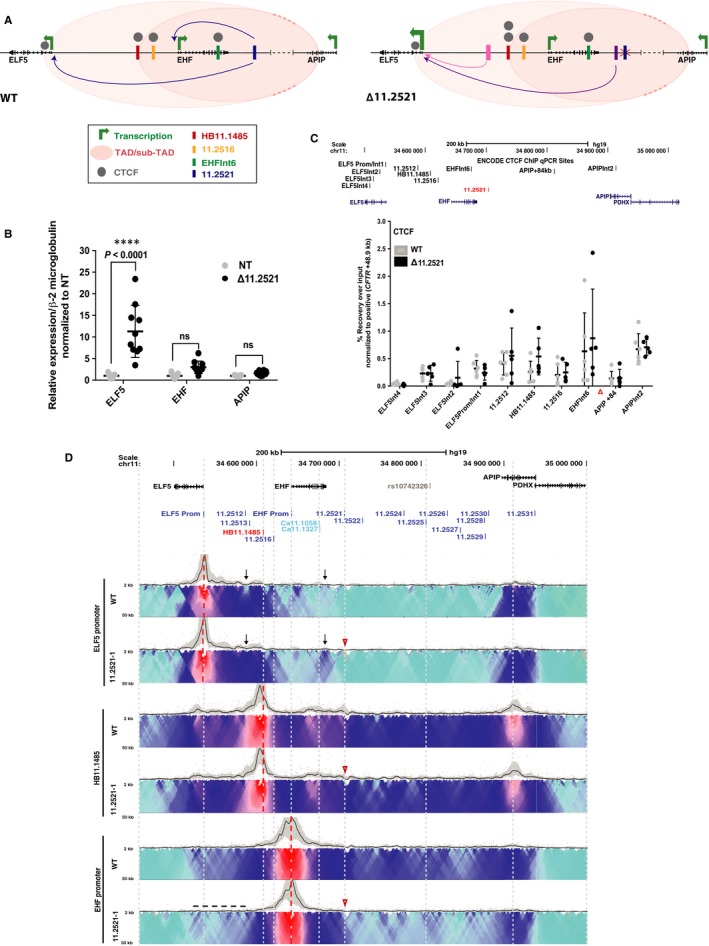
Impact of deletion of *cis*‐element 11.2521 in A549 c9 cells. (A) 11.2521 interacts with both the *ELF5* and *EHF* promoters (blue arrows) and enhances their expression. Upon deletion of 11.2521 (red X), *ELF5* expression is enhanced. Additionally, interactions between the *ELF5* promoter and DHS 11.2512/11.2513 (pink arrow) and an unknown element 3’ to the *EHF* locus (purple arrow) are evident. CTCF occupancy at 5’ sub‐TAD boundary HB11.1485 is slightly increased (two grey circles). (B) RT‐qPCR for *ELF5*, *EHF* and *APIP* expression in non‐targeted WT (NT) and deletion clones. Data are normalized to β2M and relative to clonal WT. Error bars are SD, n ≥ 3. ****P < .0001, ***P < .001, **P < .01, *P < .05, ns = not significant by a two‐way ANOVA test with multiple comparisons. (C) ChIP‐qPCR for CTCF enrichment at interacting sites across the 11p13 region in WT and deletion clones. ChIP results are shown as percent recovery over input and are normalized to a positive control (*CFTR* + 48.9 kb). Error bars are SD, n ≥ 3. (D) 4C‐seq data as in Figure 1 in WT and one 11.2521 deletion clone. Viewpoints are at *ELF5* promoter, HB11.1485 and *EHF* promoter (red dotted lines). Open red arrowheads indicate the deletion site. Informative interactions or loss thereof are shown as black dotted line or black arrows

The 11.2521 CRE was shown previously to be a strong enhancer of both the *ELF5* and *EHF* promoters (though not *APIP*), by luciferase assays in 16HBE14o‐ bronchial epithelial cells.^25^ These data were confirmed here in A549 c9 cells (Figure S1A‐B). To examine the function of this element in the genomic context, we used CRISPR/Cas9 to remove ~2.5 kb encompassing the site in A549 c9 cells. Two independent homozygous deletion clones were evaluated and compared both to non‐targeted clones and WT A549 c9 cells. Models of the 11p13 region (left) and the impact of removal of the 11.2521 CRE (right) are shown in Figure 2A. First, *ELF5*, *EHF* and *APIP* expressions were measured using RT‐qPCR (Figure 2B). Removal of this CRE did not alter *EHF* or *APIP* expression; however, it enhanced transcript abundance of *ELF5* (~11‐fold, *P* < .0001).

Since the 11.2521 CRE is an enhancer of the *ELF5* and *EHF* promoters in transient assays,^25^ the increased expression of *ELF5* upon deletion of this CRE suggests that features of the genomic architecture of the region may have a dominant role in regulating gene expression. To address this hypothesis, we measured CTCF occupancy at known binding sites across the region using ChIP‐qPCR. The 11.2521 CRE itself binds CTCF with low affinity in 16HBE14o‐ cells though not in other airway cell types.^25^ However, this element was shown earlier by 4C‐seq to interact with many other sites of CTCF occupancy across the region,^25^ so we predicted that its removal could alter occupancy at some other sites. Consistent with this prediction, removal of the 11.2521 CRE was associated with moderately elevated CTCF occupancy at HB11.1485 (~2‐fold) (Figure 2C), though this alteration did not reach statistical significance.

Next, to investigate the impact of removal of the 11.2521 CRE on chromatin architecture across chr11p13, we performed 4C‐seq on the deletion clones and compared them to WT A549 c9 cells (Figure 2D). Again, two deletion clones were analysed for each viewpoint and each clone was assayed in replica experiments. One experiment is shown in each 4C‐seq panel and is consistent with its replicates. Using a viewpoint at the *ELF5* promoter, loss of the 11.2521 CRE increased its interactions with DHS11.2512/11.2513 and an uncharacterized element immediately 3’ to *EHF* (black arrows). However, the deletion had no impact on interactions with the HB11.1485 viewpoint, while associations with the *EHF* promoter were modestly reduced (black dotted line). These data suggest that the 11.2521 CRE may inhibit *ELF5* promoter interactions with previously uncharacterized enhancer elements, and that its functions depend upon specific chromatin architecture at chr11p13.

### 3.3 Deletion of the 11.2516 CRE increases *ELF5* but not *EHF* expression and alters chromatin architecture

**Figure 3:**
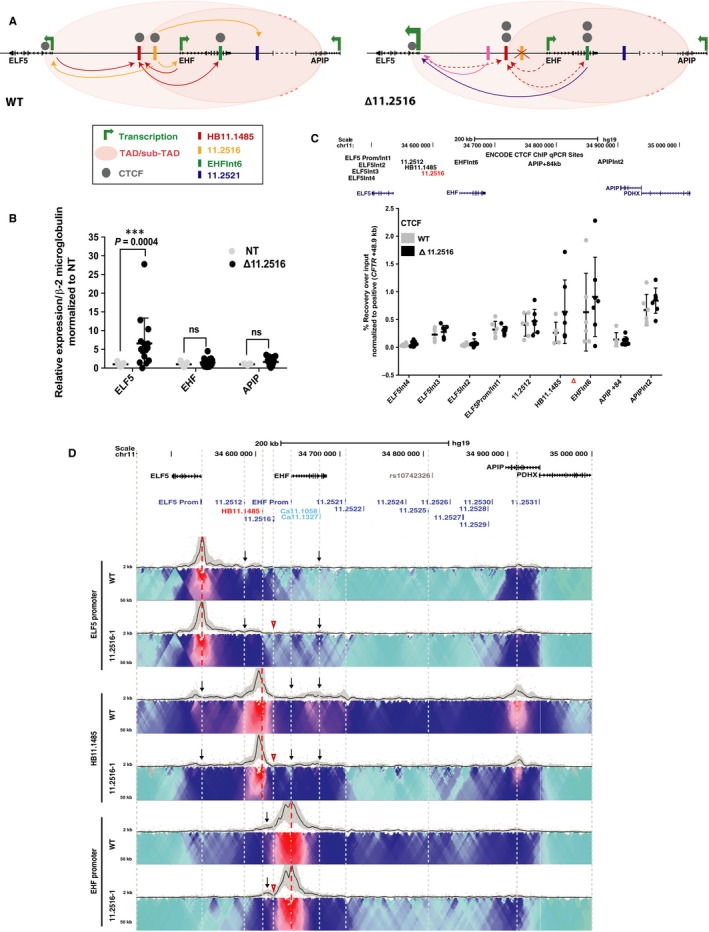
Impact of deletion of *cis*‐element 11.2516 in A549 c9 cells. (A) 11.2516 interacts with both the *ELF5* and *EHF* promoters and enhances their expression (yellow arrows). 11.2516 also interacts with the 11.2521 enhancer element (yellow arrow) and helps to recruit this DHS to the gene promoters. Furthermore, 11.2516 interacts strongly with 5’ sub‐TAD boundary HB11.1485 and helps to loop this element to the *ELF5* promoter, *EHF* promoter and *EHF* intron 6 (red arrows). Upon deletion of 11.2516 (red X), *ELF5* expression is enhanced. Additionally, interactions between HB11.1485 and the *ELF5* promoter, *EHF* promoter and *EHF* intron 6 are reduced (red dashed arrows), which allows the *ELF5* promoter to gain interactions with CREs, particularly DHS 11.2512/11.2513 (pink arrow) and *EHF* intron 6 (purple arrow). Furthermore, CTCF occupancy increases at adjacent sites HB11.1485 and *EHF* intron 6 (two grey circles). (B) RT‐qPCR for *ELF5*, *EHF* and *APIP* expression in non‐targeted WT (NT) and deletion clones. Data are normalized to β2M and relative to clonal WT. Error bars are SD, n ≥ 3. ****P < .0001, ***P < .001, **P < .01, *P < .05, ns = not significant by a two‐way ANOVA test with multiple comparisons. (C) ChIP‐qPCR for CTCF enrichment at interacting sites across the 11p13 region in WT and deletion clones. ChIP results are shown as per cent recovery over input and are normalized to a positive control (*CFTR* + 48.9 kb). Error bars are SD, n ≥ 4. (D) 4C‐seq data as in Figure 1 in WT and one 11.2516 deletion clone. Viewpoints at the *ELF5* promoter, HB11.1485 and *EHF* promoter (red dotted lines). Open red arrowheads indicate deletion site. Informative interactions are shown as black arrows

The 11.2516 CRE was also shown previously to be a strong enhancer of both the *ELF5* and *EHF* promoters (though not *APIP*), by luciferase assays in 16HBE14o‐ cells.^25^ These data were confirmed here in A549 c9 cells (Figure S1A‐B). To examine the function of this element in the genomic context, we again used CRISPR/Cas9 to remove ~2.6 kb fragment encompassing the site in A549 c9 cells. Three homozygous deletion clones were evaluated and compared to both non‐targeted clones and WT A549 c9 cells. Models of the 11p13 region (left) and the impact of removal of the 11.2516 CRE (right) are shown in Figure 3A. First, *ELF5*, *EHF* and *APIP* expressions were measured using RT‐qPCR (Figure 3B). Removal of this CRE did not alter abundance of the *EHF* or *APIP* transcripts; however, it was associated with an increase in *ELF5* expression (~7‐fold, *P* = .0004).

As for the 11.2521 CRE, it seemed probable that the in vitro enhancer activity of the 11.2516 CRE might be dominated by features of the chromatin architecture in the genomic context. To test this prediction, we measured CTCF occupancy at known sites across chr11p13 using ChIP‐qPCR. The 11.2516 CRE was shown previously to bind CTCF in multiple airway cell types.^25^ Hence, it was not unexpected to observe that removal of this element reproducibly enhanced CTCF occupancy at adjacent sites HB11.1485 (~2.4‐fold increase) and *EHF* intron 6 (~1.4‐fold increase), though these alterations did not reach statistical significance (Figure 3C). This enhancement in occupancy at nearby sites is a common feature of removal of key sites of CTCF occupancy from a genomic region.^28^, ^41^

Next, to examine the impact of removal of the 11.2516 CRE on chromatin architecture, we performed 4C‐seq on the deletion clones and compared them to WT A549 c9 cells (Figure 3D). Again, two deletion clones were analysed for each viewpoint and each clone was evaluated in replica experiments. One experiment is shown in each 4C‐seq panel and is consistent with its replicates. Removal of the 11.2516 CRE enhanced interactions between the *ELF5* promoter viewpoint and the 11.2512/11.2513 and *EHF* intron 6 elements (black arrows). These data are consistent with our previous results showing direct interaction of 11.2516 with these sites.^25^ In contrast, reduced associations were evident between a viewpoint at HB11.1485 and multiple sites across the region, including the *ELF5* promoter, *EHF* promoter and *EHF* intron 6 (black arrows). Loss of the 11.2516 CRE also coincided with a modest reduction of interactions between the *EHF* promoter and the rest of the region, with the exception of an unidentified element located between HB11.1485 and 11.2516 (black arrow), which showed an enhanced association. These data suggest that 11.2516 has an important role in the maintenance of chromatin architecture at the chr11p13 region.

### 3.4 Expression of *ELF5* is associated with altered chromatin architecture

**Figure 4:**
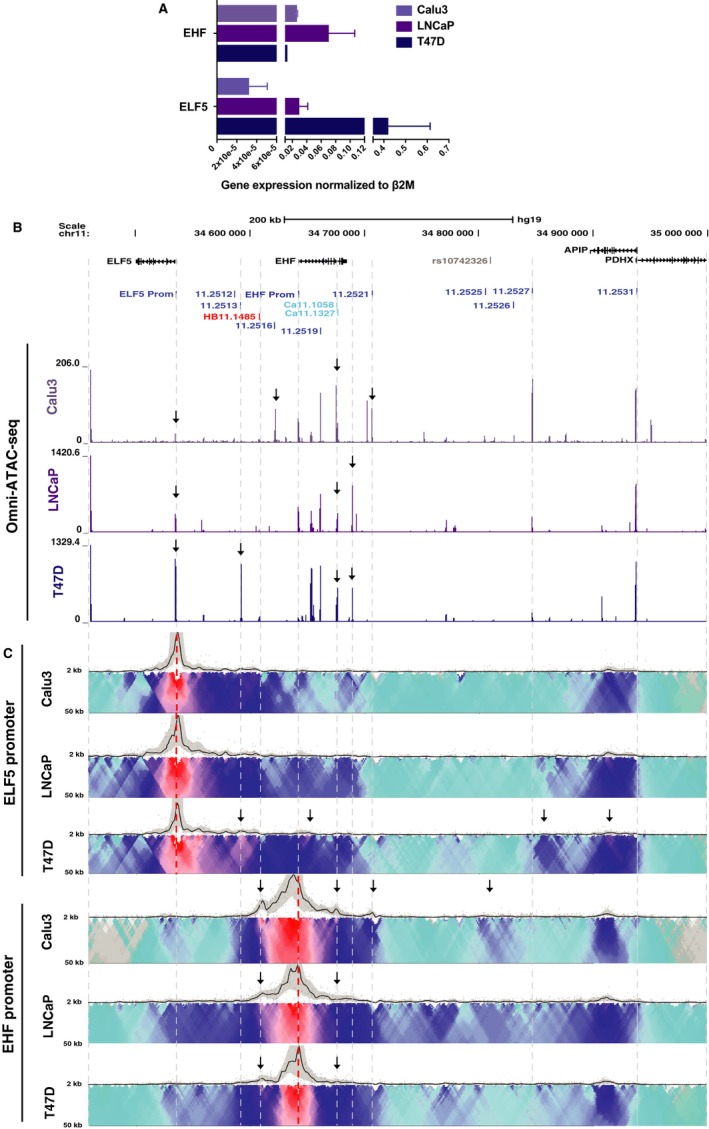
Changes in 3D organization and chromatin accessibility of 11p13 in *ELF5*‐expressing cells. (A) RT‐qPCR of *ELF5* and *EHF* expression in Calu3, LNCaP and T47D cells. Data are normalized to β2M. Error bars are SEM, n = 3. (B) ATAC‐seq showing regions of open chromatin in Calu3, LNCaP and T47D cells. Key peaks are shown by black arrows. (C) 4C‐seq data as in Figure 1 in Calu3, LNCaP and T47D cells. Viewpoints at the *ELF5* promoter and *EHF* promoter (red dotted lines). Informative interactions are shown as black arrows

None of the airway cell lines used in this work and our earlier analysis of the chr11p13 region 11, 25 express detectable levels of ELF5 protein, and *ELF5* transcript abundance is very low (Figure S2). However, primary cultures of human bronchial and tracheal epithelial cells have low and variable expression of ELF5,^42^ which may reflect the relative contribution of different cell types. Moreover, the CRISPR deletion studies described above consistently had most impact on the *ELF5* gene. Hence, it was important to examine the chr11p13 region in cell types expressing both ELF5 and EHF. The T47D (breast cancer epithelial)^24^ and LNCaP (prostate cancer epithelial) 23 cell lines met this requirement and were chosen for further study. The abundance ratio of *ELF5* to *EHF* transcripts is highest in T47D cells, and the reverse is seen in the LNCaP line (Figure 4A). To examine the correlation between *ELF5* and *EHF* expression levels and chromatin architecture at chr11p13, we performed Assay for Transposase Accessible Chromatin (ATAC)‐seq 43, 44 to map open chromatin (Figure 4B) and 4C‐seq to show 3D interactions (Figure 4C) in T47D, LNCaP and Calu3 cells (which lack *ELF5*).

Most notable are the substantially different profiles of open chromatin peaks around the *ELF5* and *EHF* loci in the different cell lines (Figure 4B). First, the relative height of the peak at the *ELF5* and *EHF* promoters correlated well with gene expression levels. A very prominent *ELF5* promoter peak is seen in T47D cells, a smaller peak in LNCaP cells and a near background peak in Calu3 cells. In contrast, both Calu3 and LNCaP cells have a similar peak of open chromatin at the *EHF* promoter, which is not strongly evident in T47D cells. ATAC‐seq data also revealed large peaks of open chromatin at the two airway enhancer elements, 11.2516 and 11.2521, which were absent in both LNCaP and T47D cells, consistent with our earlier work showing these enhancers to be airway‐selective.^25^ In transient luciferase assays in T47D cells (Figure S1C, D), the 11.2516 fragment had only modest enhancer activity on the *ELF5* and *EHF* promoters compared to airway cells. The 11.2521 element had no enhancer activity on the *ELF5* promoter in T47D cells, consistently giving lower luciferase values than the promoter alone, and again had modest enhancer effect on the *EHF* promoter. Also of note is the ATAC‐seq peak at *EHF* intron 6 in all three cell types irrespective of EHF abundance, suggesting a ubiquitous role. A novel open chromatin peak is seen close to the 3’ end of the *EHF* locus in both LNCaP and T47D cells, but not in Calu3 cells suggesting a correlation of this site with *ELF5* expression. Another new site of open chromatin is only seen in T47D cells at 11.2513, a region that corresponds to a DHS in tracheal epithelial cells^45^ but has not yet been studied.

Since expression of *ELF5* is associated with novel peaks of open chromatin at chr11p13, it seemed probable that altered chromatin architecture might also be seen in *ELF5* expressing cells. To investigate this hypothesis, we performed 4C‐seq on Calu3, LNCaP and T47D cells using viewpoints at the *ELF5* and *EHF* promoters (Figure 4C). The *ELF5* promoter viewpoint showed a similar pattern of interactions in Calu3 and LNCaP cells, with interactions coinciding with several peaks of open chromatin, both adjacent to *ELF5* and across the *EHF* locus and with the 3’ TAD boundary in *APIP* intron 3. In contrast, high *ELF5* expression in T47D cells coincided with strong interactions (black arrows) between the *ELF5* promoter viewpoint, the novel peak of open chromatin at 11.2513 and a site in *EHF* intron 1, also associated with an ATAC‐seq peak. Interactions with the 3’ TAD boundary and an element 3’ of 11.2527 were also seen.

Using the *EHF* promoter viewpoint, the major specific difference seen in Calu3, LNCaP and T47D cells was a great reduction in interactions with the HB11.1485 5’ sub‐TAD boundary when the *ELF5* gene is highly expressed (T47D) (black arrows). A minor reduction of interactions with the *EHF* intron 6 CRE was also observed in LNCaP and T47D cells (black arrows). In addition, in Calu3 cells only, interactions were evident with the airway enhancers (11.2516 and 11.2521), *EHF* intron 6 and the 11.2525/11.2526 sites closest to the highest p‐value SNP (rs10742326) from the GWAS^4^ (black arrows). In summary, these data suggest that alterations in chromatin architecture at chr11p13 and the loss of a 5’ sub‐TAD boundary enable expression of *ELF5*.

### 3.5 Activation of CREs across chr11p13 enhances *ELF5* expression, but has little impact on *EHF* or *APIP*

**Figure 5:**
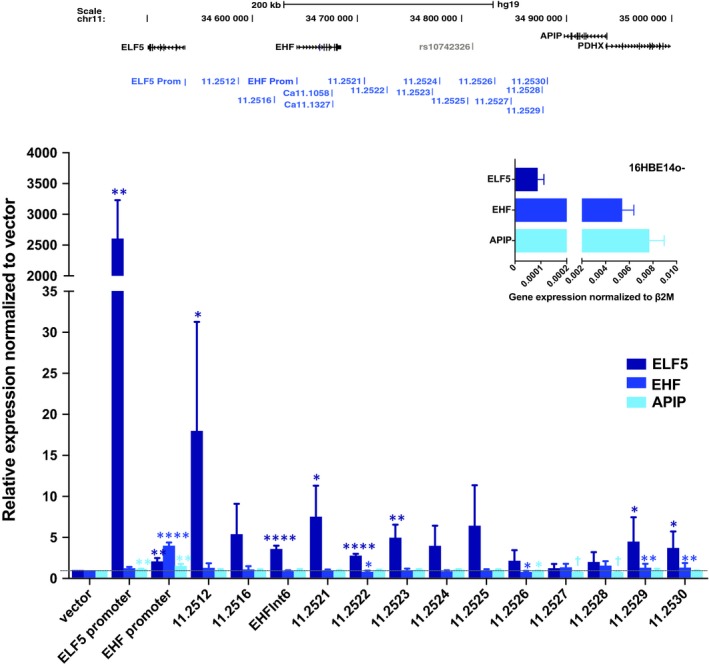
Changes in 11p13 gene expression with VPR‐mediated activation of promoters and *cis*‐elements in 16HBE14o‐ cells. VPR‐mediated activation of *ELF5* promoter, *EHF* promoter and DHS at 11p13. *EHF* intron 6 encompasses Ca11.1058 and Ca11.1327. Error bars are SEM, n = 3. ****P < .0001, ***P < .001, **P < .01, *P < .05, ns = not significant by an unpaired Student's t test. † *P* < .0001 – statistically significant, but not biologically significant due to low expression levels. Inset panel shows *ELF5*, *EHF* and *APIP* gene expression for 16HBE14o‐ cells. Error bars are SEM, n = 3

The data shown in the previous sections suggest that in cells that express *ELF5* this locus may have an important role in the functional genomics of the chr11p13 region. Our earlier in vitro studies suggested that the 11.2516 and 11.2521 CREs enhanced activity of both the *ELF5* and *EHF* promoters 25; however, results presented here suggest that other features of the chromatin may have a dominant effect in vivo. To determine whether activation of any CREs across chr11p13 could elevate endogenous gene expression, we used CRISPRa. Briefly, nuclease‐null dCas9 was used in the dCas9‐VPR fusion, which recruits three transcriptional activators, VP64 (a transcriptional activator made from 4 tandem copies of the Herpes Simplex Viral Protein 16), NF‐kappa‐B *p65* subunit [p65], and Replication and transcriptional activator [Rta] (SP‐dCas9‐VPR) to target sites. A minimum of two guide RNAs were designed to target the *ELF5* and *EHF* promoters and multiple airway DHS across chr11p13 in 16HBE14o‐ and A549 cells (Figure 5 and Figure S3). Targeting the *ELF5* promoter increased its expression by ~2600‐fold in 16HBE14o‐ and ~40‐fold in A549 cells. Targeting the *EHF* promoter increased its expression by ~4‐fold in 16HBE14o‐ and ~2‐fold in A549 cells. Among targeted DHS, 11.2512, which we had not studied previously, resulted in the most robust activation of *ELF5* (~18‐fold in 16HBE14o‐ cells and ~7‐fold in A549). Expression of *ELF5* in 16HBE14o‐ cells was also increased significantly by CRISPRa at the *EHF* intron 6 (~3.5‐fold), 11.2521 (~7.5‐fold), 11.2522 (~3‐fold), 11.2523 (~5‐fold), 11.2529 (~4.5‐fold) and 11.2530 (~4‐fold) DHS (Figure 5). Activation of most of the same sites together with 11.2516 also significantly increased *ELF5* expression in A549 cells (Figure S3). *APIP* expression was not altered by activation of any of the DHS in 16HBE14o‐ cells, while only targeting the 11.2529 and 11.2530 sites slightly, but significantly, increased *EHF* expression (~1.3‐fold). The modest response to CRISPRa at chr11p13 suggests that either additional sequences, which are not associated with regions of open chromatin in airway cells, are key in transcriptional activation of *ELF5* and *EHF*, or that a more complex regulatory mechanism exists for the genes in this region. The latter explanation would be consistent with our 4C‐seq observations suggesting close interactions between the *ELF5* and *EHF* genes and their CREs.

### 3.6 ELF5 occupies regions of open chromatin at *EHF* and represses its expression

**Figure 6:**
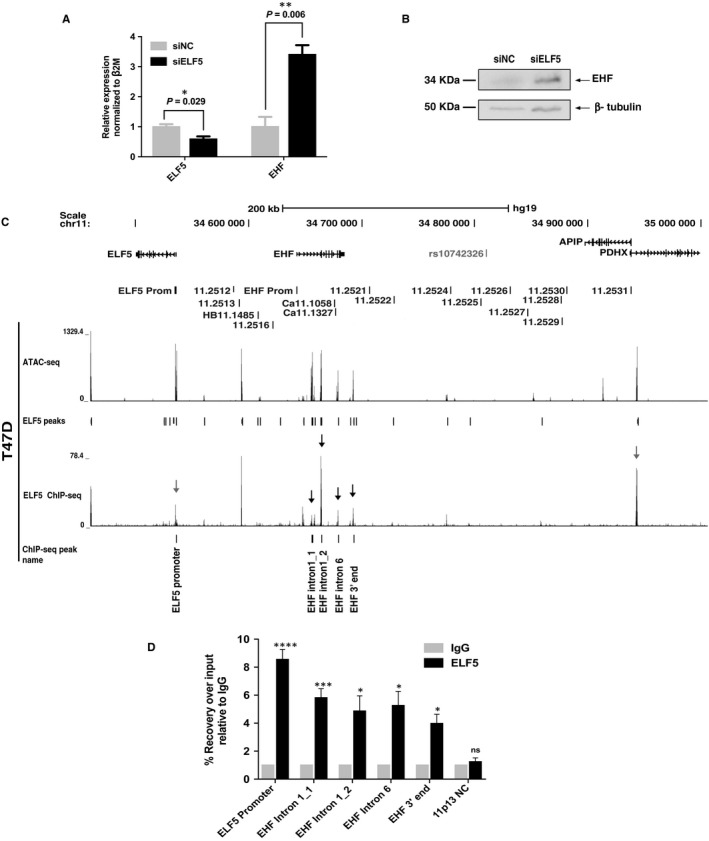
ELF5 binds to *EHF* locus and represses gene expression. (A) mRNA expression analysis of EHF and ELF5 during siRNA‐mediated depletion of ELF5. Error bars are SEM, n = 3. ****P < .0001, ***P < .001, **P < .01, *P < .05, ns = not significant by an unpaired Student's t test. (B) Western blot showing increase in EHF protein levels in response to transfection of T47D cells with siRNA against ELF5. (C) Open chromatin and ELF5 occupancy across 11p13 in T47D cells; ATAC‐seq analysis, upper track and sites of ELF5 occupancy identified by ELF5 ChIP‐seq, lower track. (D) ChIP followed by qPCR analysis confirming enrichment of ELF5 at multiple sites (marked) within the *EHF* locus. Error bars are SEM, n = 3. ****P < .0001, ***P < .001, **P < .01, *P < .05, ns = not significant by an unpaired Student's *t* test

We next tested the hypothesis that the close physical interactions between the active *ELF5* and *EHF* loci and their CREs might underlie the complex regulatory environment at chr11p13. We first performed siRNA‐mediated depletion of ELF5 in T47D cells, which express ELF5 and EHF, and assayed expression of both genes by RT‐qPCR. Primers located in exons 5 and 6 for *ELF5* and exons 3 and 4 for *EHF* (Table S3) enabled detection of all major isoforms of both transcripts. Our results show that ELF5 depletion significantly increases EHF mRNA expression (Figure 6A). Correspondingly, ELF5 depletion also significantly increased EHF protein expression, suggesting that *EHF* transcription is repressed by ELF5 (Figure 6B).

A previous study^18^ suggested a direct repression of *EHF* by ELF5, though as ELF5 was overexpressed, there were concerns of potential off‐target effects of the TF. To determine whether the repression of *EHF* by ELF5 observed in our experiments was dependent on direct occupancy of CREs for the *EHF* locus by ELF5, we performed ChIP‐seq using an antibody specific for endogenous ELF5 in T47D cells. The Irreproducible Discovery Rate (IDR) analysis of two replica experiments is shown in Figure 6C and reveals multiple sites of ELF5 occupancy across the *EHF* locus, most notably in introns 1 and 6 and close to its 3’ end (black arrows). Each of the peaks of ELF5 binding also coincides with a peak of open chromatin in T47D cells, mapped by ATAC‐seq (Figure 6C upper track). The sites of ELF5 occupancy at the *EHF* locus were confirmed by ChIP‐qPCR analysis (Figure 6D). In addition, ELF5 occupancy coinciding with sites of open chromatin is seen at the *APIP*/*PDHX* and *ELF5* gene promoters (grey arrows), suggesting that ELF5 may have a major impact across 11p13, both through controlling other genes and by autoregulation. These results are highly relevant to primary bronchial and tracheal epithelial cells, which also express both ELF5 and EHF, albeit with much lower ELF5 abundance than in T47D cells.

## 4 DISCUSSION

Intense interest in the chr11p13 region arose through its association with lung disease severity in CF; however, the genetic mechanisms underlying the functions of this region are not well understood. Our data reveal a complex regulatory network at chr11p13 that coordinates the expression of the ETS transcription factors ELF5 and EHF. We previously identified several putative airway epithelial cell‐selective enhancer elements of *ELF5* and *EHF* within the region and defined important aspects of the chromatin architecture relevant to the regulation of genes at chr11p13.^25^ Here, we performed functional genomics experiments to elucidate the endogenous role of two predicted enhancers and a CRE that lies within a stretch enhancer at the *EHF* locus.

Deletion of the CRE in *EHF* intron 6 and the two airway epithelial cell‐selective enhancers at 11.2516 and 11.2521 using CRISPR/Cas9 protocols had unexpected effects on gene expression, and were associated with modest changes in CTCF occupancy and local higher order chromatin structure. Firstly, removal of the two enhancers that were shown to activate the *ELF5* and *EHF* gene promoters in transient luciferase assays^25^ did not reduce endogenous *ELF5* or *EHF* expression. Removal of either element enhanced ELF5 transcript abundance and had no impact on EHF levels. Though these data do not support a simple enhancer role for the 11.2521 and 11.2516 CREs, these elements are both marked with active histones (H3K27Ac) and furthermore lack repressive marks in several airway cell types, supporting their role as enhancers in the genomic context.^25^ This suggests additional mechanisms contribute to *ELF5* and *EHF* gene expression levels in their endogenous location, perhaps providing functional redundancy. These factors may include features of the higher order chromatin structure and organization. This interpretation would be consistent with the luciferase assays showing enhancer activity, since within the reporter gene constructs potential CREs are sited next to a gene promoter without consideration for architectural barriers such as CTCF sites. It is also possible that eRNA transcription arising at these CREs may repress nearby gene expression.^46^ Global run‐on sequencing (GRO‐seq) in HCT116 cells shows eRNA transcription at the three of CREs studied here, notably from the antisense strand at *EHF* intron 6 (Figure S4).^47^ This CRE may repress *EHF* transcription, as intragenic eRNA expression is known to interfere with Pol II‐mediated transcription,^46^ in which case removal of the element could enhance EHF expression. The CRISPR/Cas9 mediated deletion experiments also uncovered a novel and important function for the CRE at *EHF* intron 6. This element appears to facilitate looping of the nearby enhancer, 11.2516, to both the *ELF5* and *EHF* promoters. The observed reduction in CTCF occupancy at 11.2516 (Figure 1C), accompanied by loss of the 11.2516 interactions with the promoters as shown by 4C‐seq (Figure 1D), upon deletion of intron 6 is supportive of this role. These data are consistent with the reported mechanisms of looping of enhancers to target promoters at other loci.^28^, ^48^, ^49^, ^50^ In summary, the removal of key CREs from chr11p13 did not add clarity to our understanding of its functional genomics. However, importantly it focused our interest on the potential role of *ELF5* in regulatory mechanisms in the region, since this gene was the most responsive to CRE manipulations.

ELF5 is absent from commonly used human airway epithelial cell lines, though it is expressed at low levels in primary HBE and HTE cells.^42^ Hence, in order to pursue our interest in ELF5 and the 5’ end of the chr11p13 region we used other epithelial cell lines (T47D and LNCaP) that express both *ELF5* and *EHF*. Analysis of these lines revealed both novel peaks of open chromatin (probable CREs) and an altered chromatin architecture associated with expression of *ELF5*. Two sites are of particular interest: 11.2513, is a peak of open chromatin in primary HTE cells^45^ but lacks H3K27ac in the airway cell types^25^ so was not investigated further; also of interest is a novel element immediately 3’ to the *EHF* locus, which is not evident in primary HTE cells, but is clearly seen in both *ELF5*‐expressing cell types studied here. Upon removal of the 11.2521 enhancer from airway cells, the *ELF5* promoter gains interactions with this element (Figure 2D) suggesting that this site is at least in part, responsible for the large increase in *ELF5* transcript levels in these deletion clones. It seems probable the 11.2513 and novel CREs either harbour enhancers of the *ELF5* promoter and/or are sites of occupancy for this transcription factor.

In addition to the novel CREs observed in T47D and LNCaP cells, 4C‐seq data showed a loss of interactions between the *EHF* promoter and the HB11.1485 CRE in comparison to the airway cell lines (Figure 4C). In this context, our earlier work^25^ where we noted that though HB11.1485 is a CTCF binding site that may demarcate a sub‐TAD boundary in airway cells, it might also have a structural role in the cell type‐specific regulation of *ELF5*, was prescient. The loss of interaction between the *EHF* promoter and HB11.1485 in *ELF5‐*expressing cells suggests this site is a key sub‐TAD boundary between the *EHF* and *ELF5* promoters restricting their recruitment of nearby cell‐type specific enhancers. Upon expression of *ELF5,* this sub‐TAD boundary may disappear to enable interactions between the *ELF5* promoter and the CREs at the 5’ end of chr11p13.

Our new data on the role of CREs 5’ to the *EHF* locus in *ELF5* expressing cells, and the 4C‐seq results showing direct interactions of the *ELF5* promoter with elements within *EHF*, suggested that a potential regulatory relationship between these TFs warranted further study. Our results showed that ELF5 directly regulates the *EHF* gene. Using ChIP‐seq for ELF5 in T47D cells, with validation of ELF5 occupancy by ChIP‐qPCR we identify multiple binding sites for ELF5 within the *EHF* locus, particularly within introns 1 and 6. These sites of ELF5 occupancy coincide with peaks of open chromatin, which are particularly evident in *ELF5*‐expressing cells. Furthermore, siRNA‐mediated depletion of the *ELF5* transcript enhanced EHF mRNA and protein levels. These data suggest that ELF5 is a direct repressor of *EHF* expression. Our results are consistent with observations on other ETS factors, which are known to regulate each other (reviewed in^51^); however, the identification of an ETS family member regulating the important EHF TF is novel.

Returning to the role of chr11p13 as a modifier of lung disease severity, we do not address here the mechanisms underlying the association of high p‐value SNP close to the chr11.2525 site. However, our observations on cell‐specific recruitment of CREs across the modifier region and most importantly on the direct regulation of *EHF* by ELF5 are significant advances in understanding the region. EHF is intimately involved in regulating the wound response, inflammation and tissue remodelling, all of which are integral components of CF lung pathology.^11^, ^12^ For example, EHF enhances expression of S100 Calcium Binding Proteins A8 and A9 (*S100A8* and *S100A9*), which are essential for cell motility^52^, ^53^, ^54^ and thus the wound response. Proper wound response is crucial for injury repair, and this process is delayed in the CF lung.^12^ Also, EHF enhances the transcription of genes encoding the pro‐inflammatory cytokines interleukin 8 (IL‐8) and C‐X‐C motif chemokine ligand 6 (CXCL6).^11^, ^12^ These secreted cytokines are necessary for the recruitment of neutrophils to a site of infection or injury.^55^, ^56^ Increasing the neutrophil burden in the CF lung can lead to chronic inflammation and eventual tissue remodelling,^57^ both of which significantly contribute to patient mortality. Furthermore, EHF promotes expression of Sam Pointed‐Domain Containing ETS Transcription Factor (*SPDEF*),^12^ which is involved in interleukin 13 (IL‐13)‐mediated goblet cell differentiation.^58^ Enhanced goblet cell differentiation increases expression of the mucin 5AC (*MUC5AC*) gene,^59^, ^60^ which encodes one of the major secreted mucins in the lung (reviewed in^61^) and is a hallmark of CF lung disease. Since our data show that ELF5 occupies open chromatin sites at the *EHF* locus and represses its expression, it is likely that this ETS factor also plays an important role in lung biology. Modulation of *ELF5* expression would be predicted to impact many of the EHF‐mediated processes discussed above. Although EHF remains the primary candidate for influencing lung phenotype differences observed in CF patients, ELF5 likely also plays an important role through its regulation of *EHF* expression. These observations are not only relevant to CF but also to other airway diseases associated with inflammation, defective wound response and lung tissue remodelling, including asthma, chronic obstructive pulmonary disease (COPD) and bronchiectasis.^62^, ^63^

## CONFLICTS OF INTEREST

The authors confirm that there are no conflicts of interest.

## AUTHOR CONTRIBUTIONS

HS, JSE, KML and AH conceived and designed the project. HS, JSE, KML, SY, JLK, MN, CC, EMM and S‐HL designed and performed experiments. HS and AH wrote the manuscript.

## Data Availability

Genome‐wide data are deposited at GEO: GSE131084.
