# Supplementary material for: Coordinate regulation of ELF5 and EHF at the chr11p13 CF modifier region
Source: J Cell Mol Med. 2019 Sep 26;23(11):7726–40. doi: 10.1111/jcmm.14646 (PMC6815777; doi:10.1111/jcmm.14646)
Supplement: Supplementary file 1 [file JCMM-23-7726-s001.pdf]

Supplemental Table S1: gRNA sequences used to generate CRISPR/Cas9 deletion clones.

	5'	3'
**EHF intron 6 gRNAs**	GTCAATAGTCCTTCTTAAGAT	GTTTAGGTCGTGGAATCAGAG
**11.2521 gRNAs**	GTTTCCATGCCTCTTACCAT	GTAAGGATATGCTGGTAGGA
**11.2516 gRNAs**	GAGTGTTTCTGATGATCAGTG	GACAGAGCTGGCATAGTAAAT

Supplemental Table S2: PCR primers used for validation of CRISPR/Cas9 homozygous deletion clones.

	Forward	Reverse
**EHF intron 6 PCR (external)**	AGCTGGGCCATCCTCTTTTC	TTTTTGGTGTGGCACTTGGC
**EHF intron 6 PCR (internal/external)**	CTCCATCCTGCCATGACGTT	TTTTTGGTGTGGCACTTGGC
**11.2521 PCR (external)**	GGCGATGTGTTCAAAGTGCT	TGGGTAGGGAACTGGGTAGG
**11.2521 PCR (internal/external)**	GGCGATGTGTTCAAAGTGCT	TTACCCAACCCCACAGGAGA
**11.2516 PCR (external)**	GTACAGGGGCCACACATAGC	GCAGGGTGCTGCTTTACAGA
**11.2516 PCR (internal/external)**	GAGGTGGTTGCTCCAACGTA	GCAGGGTGCTGCTTTACAGA

Supplemental Table S3: Primers used for RT-qPCR.

	Forward	Reverse
**Beta-2 -Microglobulin (β2M)**	ACTGAATTCACCCCCACTGA	CCTCCATGATGCTGCTTACA
**ELF5**	TGCTTGAAAACAAGTGGCATC	AGGGCTTCCGATTTAACCACC
**EHF**	GCAGCATGAGTTTGCAGGAG	GTGTGTGGACTGGAAACAGGT
**APIP**	GGCCACACTTCTCTTTCCAG	GCATGAGCCATTCTATCTTTGAGG

Supplemental Table S4: Primers used for ChIP-qPCR.

	Forward	Reverse
**11p13 Negative Control**	TCCTTCCAGGTTTTGGCTCC	GCCCCAGATCAGGAGAGAGA
**CFTR +48.9**	GGCATCAGCCAGTCAAGGTT	AGCAGAGGGCAAAGTGGTACTT
**ELF5 Intron 4**	CCCCATGACACTAGCAGTCC	TACAGTCCTGGGTTCACTGC
**ELF5 Intron 3**	ACTCGCTCCCTTTTCCCTAA	AAGTCCTTTCCCATCGTGTG
**ELF5 Intron 2**	CTGAATGTCTAAGACTTGGTTGGA	TTAAAGAATGAGGTTAACAGAAGTGA
**ELF5 Promoter/Intron 1**	AGAGGGCAAATGGGTTTCTT	CACCCCCTCGATACAGAGAA
**11.2512**	GCTTCTATTCATTCACCCAACAC	GTAGTAGCCCTGCCACCAGA
**HB11.1485**	GATTTTCCGAAGCTGTGGAGG	CCACCATACGCAATCACAGG
**11.2516**	AATCTCTTCCTGCCCCACCT	TCCGGTAGGGGGTAATGACA
**EHF Intron 6**	CCCGTAAAGAAATGGCTCAC	AGGCCAAGGTCCTATCCAGT
**APIP +84 kb**	TCATGAGAAGCCACATGGAG	GACATTTCTGGTGCCTTGGT
**APIP Intron 2**	CTGTCATGCAAAGAATCAGGTTT	TAAGAAACTGTCCAGCAGAGGTC

**ELF5 ChIP Peak 1**	CACAGAAACCCCACACACTG	TAGTTTGGGGCATTTTCAGG
**ELF5 ChIP Peak 2**	CCCAGCAGTCTTTTCTCTGC	CCTGCGTTTTCAGTTTGGAT
**ELF5 ChIP Peak 3**	AAGCAGGGGAACTGGAATCT	CTTCCAGGCTTAGGGAGGAC
**ELF5 ChIP Peak 4**	CTGAAATAATGGCCCTGCAT	TGTAGGTTGCAGGGTGAGGT
**ELF5 ChIP Peak 5**	GGTGCCATCTCTCTGAAAGC	ACGTGTCCTCAGGTCAAACC

Supplemental Table S5: Primers used for 4C library generation. Red indicates P5 sequence; blue indicates P7 sequence.

	Reading Primer	Non-Reading Primer
**ELF5 Promoter**	AATGATACGGCGACCACCGAACACTCTTT CCCTACACGACGCTCTTCCGATCTGCCTC CTTTCAAGCTTTAAAATTC	CAAGCAGAAGACGGCAT ACGAAATCCTCTCACGAA CTGCC
**HB11.1485**	AATGATACGGCGACCACCGAACACTCTTT CCCTACACGACGCTCTTCCGATCTCTTGG GGAGTAGCAAAAGAT	CAAGCAGAAGACGGCAT ACGATCCAAACCTCTATT TCCTCA
**11.2516**	AATGATACGGCGACCACCGAACACTCTTT CCCTACACGACGCTCTTCCGATCTCTCCC CAAATTAGCACCATG	CAAGCAGAAGACGGCAT ACGAAGGCAGCCTTCTT GCTTTCT
**EHF Promoter**	AATGATACGGCGACCACCGAACACTCTTT CCCTACACGACGCTCTTCCGATCTTTTAGT CCACCCTGCTTTGG	CAAGCAGAAGACGGCAT ACGATTAGGGCTCAGAG TACACGG

Supplemental Table S6: sgRNAs used for VPR-mediated activation. Red indicates BbsI (BpiI) cut site.

	Forward	Reverse
**ELF5 Promoter**	CACCGCACAGCCCCTTTGATGA	AAACTCATCAAAGGGGCTGTGC
**EHF Promoter**	CACCGTAGGGAATTCCCGGTTGG	AAACCCAACCGGGAATTCCCTAC
**11.2512**	CACCGTCCTTTCTGGAGACAATAGG	AAACCCTATTGTCTCCAGAAAGGAC
**11.2516**	CACCGTTCAAAGACAGATGATGGGG	AAACCCCCATCATCTGTCTTTGAAC
**EHF Intron 6**	CACCGTCTACACTAGATAAACCATG	AAACCATGGTTTATCTAGTGTAGAC
**11.2521**	CACCGATGCGAGGTATTCTTCCCGG	AAACCCGGGAAGAATACCTCGCATC
**11.2522**	CACCGATGTCTCAGAGTCTCAACCA	AAACTGGTTGAGACTCTGAGACATC
**11.2523**	CACCGCCACCATTAGCTGTGCAGTG	AAACCACTGCACAGCTAATGGTGGC
**11.2524**	CACCGTCATGCATTGCTTAACAACA	AAACTGTTGTTAAGCAATGCATGAC
**11.2525**	CACCGCTTACCTCCACAGCACACAC	AAACGTGTGTGCTGTGGAGGTAAGC
**11.2526**	CACCGACTGTTTATTGCAGATAAGG	AAACCCTTATCTGCAATAAACAGTC
**11.2527**	CACCGTCAGCCCCTGACTCCCACTG	AAACCAGTGGGAGTCAGGGGCTGAC
**11.2528**	CACCGAAGGTGTTTCACAATCTGAG	AAACCTCAGATTGTGAAACACCTTC
**11.2529**	CACCGAAGATTTGGCTTGGCTTCAG	AAACCTGAAGCCAAGCCAAATCTTC
**11.2530**	CACCGAAAGTGAGGTCATGATTCAG	AAACCTGAATCATGACCTCACTTTC
