# Supplementary material for: Coordinate regulation of ELF5 and EHF at the chr11p13 CF modifier region
Source: J Cell Mol Med. 2019 Sep 26;23(11):7726–40. doi: 10.1111/jcmm.14646 (PMC6815777; doi:10.1111/jcmm.14646)
Supplement: Supplementary file 2 [file JCMM-23-7726-s002.pdf]

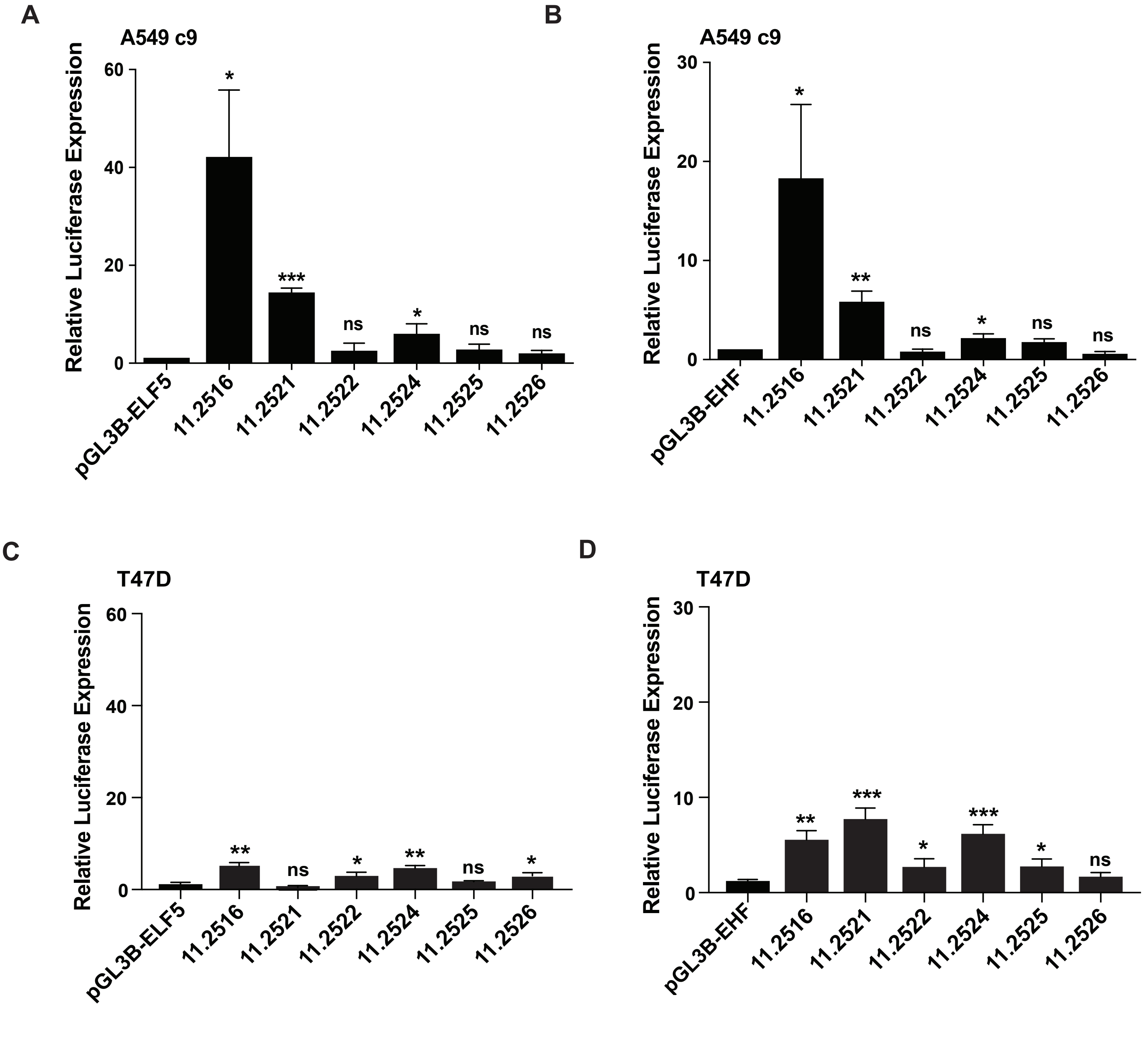

Suppl. Figure 1. The 11.2516 and 11.2521 DHS encompass airway-selective enhancer elements of *ELF5* and *EHF*. Open chromatin peaks from 11p13 were inserted into the enhancer sites for the **(A,C)** pGL3B-*ELF5* and **(B,C)** pGL3B-*EHF* promoter constructs and transfected into A549 c9 **(A,B)** and T47D **(C,D)** cells with a Renilla vector as transfection control. Luciferase expression is normalized to Renilla values, n=3. For all panels, ****p<0.0001, ***p<0.001, **p<0.01, *p<0.05, ns = not significant.

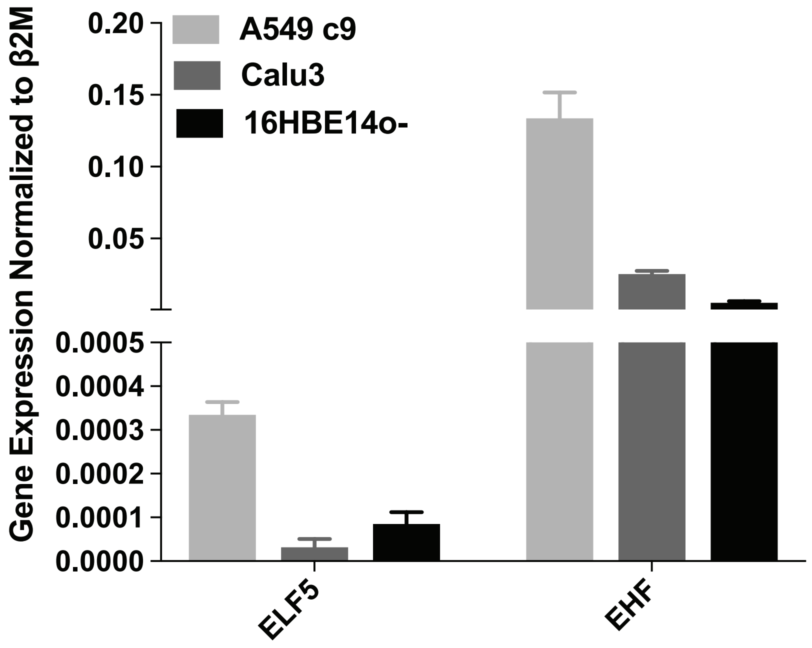

Suppl. Figure 2. Relative expression levels of *ELF5* and *EHF* in different airway cell types. The expression of *ELF5* and *EHF* were quantified using SYBR Green qPCR assays and analyzed relative to β-2-microglobulin (β2M). Data are shown for A549 c9 (light gray), Calu3 (medium gray) and 16HBE14o- (black) cells. Error bars are SEM , n=3.

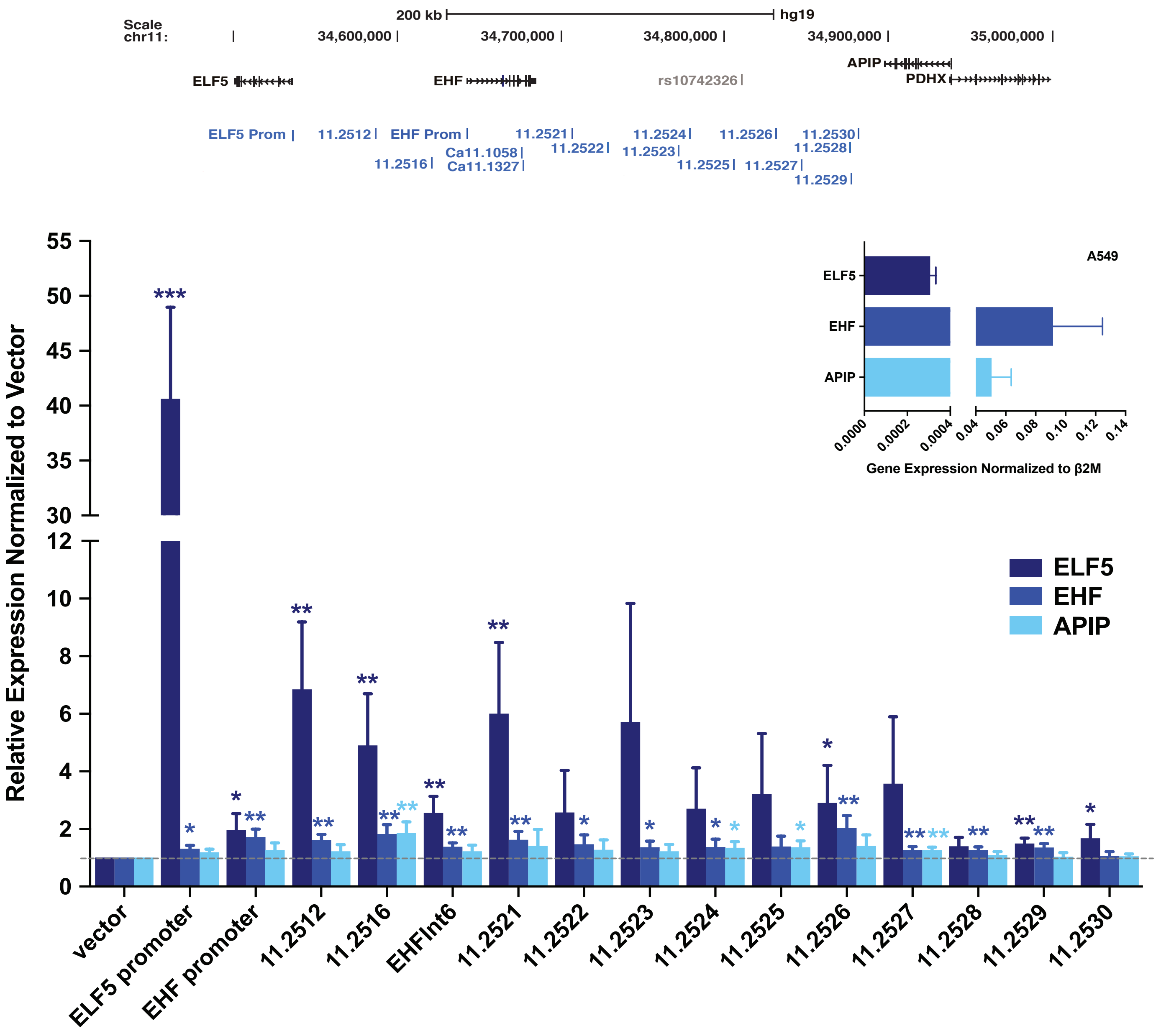

Suppl. Figure 3. Changes in 11p13 gene expression with VPR-mediated activation of promoters and *cis*-elements in A549 cells. VPR-mediated activation of *ELF5* promoter, *EHF* promoter and DHS at 11p13. *EHF* intron 6 encompasses Ca11.1058 and Ca11.1327. Error bars are SEM, n=3. ****p<0.0001, ***p<0.001, **p<0.01, *p<0.05. Upper right panel shows *ELF5*, *EHF* and *APIP* gene expression for A549 cells. Error bars are SEM , n=3.

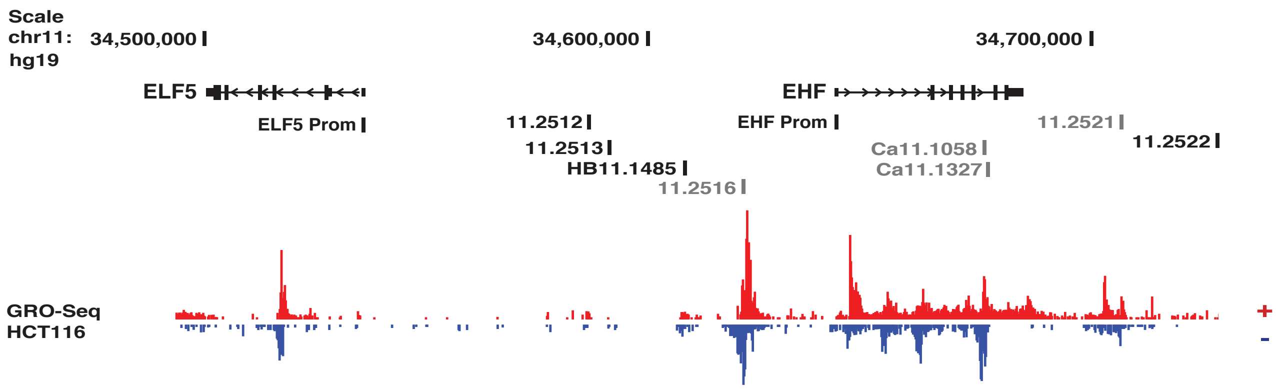

Suppl. Figure 4. Enhancer RNA (eRNA) expression from CREs at 11p13. GRO-seq data in HCT116 cells showing eRNA transcript from sense (red) and antisense (blue) strands [47]. 11.2516, *EHF* intron 6 (Ca11.1058 and Ca11.1327) and 11.2521 CREs are shown in gray.
